# Ultrasonic High-Resolution Imaging and Acoustic Tweezers Using Ultrahigh Frequency Transducer: Integrative Single-Cell Analysis

**DOI:** 10.3390/s23041916

**Published:** 2023-02-08

**Authors:** Hayong Jung, K. Kirk Shung, Hae Gyun Lim

**Affiliations:** 1 Department of Biomedical Engineering, University of Southern California, Los Angeles, CA 90089, USA; 2 Department of Biomedical Engineering, Pukyong National University, Busan 48513, Republic of Korea

**Keywords:** ultrasound, B-mode imaging, single-cell imaging, acoustic tweezer, ultrahigh frequency transducer

## Abstract

Ultrasound imaging is a highly valuable tool in imaging human tissues due to its non-invasive and easily accessible nature. Despite advances in the field of ultrasound research, conventional transducers with frequencies lower than 20 MHz face limitations in resolution for cellular applications. To address this challenge, we employed ultrahigh frequency (UHF) transducers and demonstrated their potential applications in the field of biomedical engineering, specifically for cell imaging and acoustic tweezers. The lateral resolution achieved with a 110 MHz UHF transducer was 20 μm, and 6.5 μm with a 410 MHz transducer, which is capable of imaging single cells. The results of our experiments demonstrated the successful imaging of a single PC-3 cell and a 15 μm bead using an acoustic scanning microscope equipped with UHF transducers. Additionally, the dual-mode multifunctional UHF transducer was used to trap and manipulate single cells and beads, highlighting its potential for single-cell studies in areas such as cell deformability and mechanotransduction.

## 1. Introduction

Ultrasound imaging is recognized for its advantages over other biomedical imaging techniques, including its high spatial and temporal resolutions [1,2,3,4]. The technology is non-invasive, real-time, and cost-effective [5,6,7,8]. The development of acoustic scanning microscopy dates back to the early 1970’s, when it was first invented by Lemons and Quate at Stanford University [9]. Since then, it has been developed into a valuable tool for non-destructive analysis of biological tissues and for ultrasonic imaging with a resolution of a few micrometers [9,10,11,12,13,14,15,16,17,18].

The imaging resolution in ultrasound imaging is determined by several factors, including the propagation speed of the waves, the structure of the transducer, its focal number, and the operating frequency. The operating frequency is a crucial factor that has a direct impact on the imaging resolution. Higher operating frequencies result in higher-resolution ultrasonic images. The advancements in fabrication technology and beam-forming techniques have opened up new possibilities for ultrasonic imaging that were previously not feasible due to technical limitations [19,20,21,22,23,24]. The improvement in hardware performance, such as data processing speed and sampling speed, has also contributed to the advancement of ultrasound imaging. This led to various modifications of the imaging technique, including the use of microbubble contrast agent, elastography, applied radiation force imaging, and shear wave imaging [25,26,27,28,29,30,31,32].

With the growth of technology, high frequency single element and array transducers have been developed [33,34,35,36,37,38]. In particular, the development of an ultrahigh frequency (UHF) transducer over 100 MHz has led to improved ultrasound imaging technologies due to its superior resolution (less than 20 μm) with a low f-number [39]. Recently, UHF linear arrays utilizing ZnO thin film have been fabricated, and the imaging of a zebrafish eye using 101 MHz self-focused ZnO transducers was reported [40,41].

The development of higher frequency transducers has also expanded the application of ultrasound technology from tissue studies to single-cell studies. Briggs et al. [42] utilized a UHF acoustic scanning microscope to study the contrast mechanism with MCF-7 and Hela cells with the goal of measuring the mechanical properties of cells. To improve the contrast, researcher used a phased array beam-forming and dynamic apodization method [18]. Czarnota and Kolios characterized the changes in cell death using a quantitative ultrasound method, focusing on the nucleus and configuration of the cell’s morphology. Their hypothesis was that the ultrasound signal detected during the apoptosis process would be related to backscattering intensity and frequency dependence. Their results showed that changes in cell morphology were associated with changes of a cell’s acoustic property [43]. Moore and Kolios have combined ultrasound technology with photonics to image a single leukocyte. Their results suggest that multi-modality imaging, which compensates for each other’s limitation, can enhance imaging resolution and provide a more powerful single-cell diagnosis [44].

In this paper, we employed a multifunctional UHF transducer for cell imaging and acoustic tweezer applications. The UHF transducer was designed to support a low f-number with a press-focused method, resulting in a resolution less than 10 μm without the need for an additional lens. The high operating frequency of the UHF transducer enables high-resolution cell imaging. The experimental setup was established with PC-3 cells and verified using acoustic scanning techniques. Additionally, the same UHF transducer was used to demonstrate its capability for trapping single PC-3 cells using an acoustic tweezer technique, without damaging it. Acoustic tweezers are an emerging tool in the field of biomedical and physical research [45,46,47,48] due to their non-contact and strong radiation forces [49,50]. They have been widely used for single-cell analysis, such as measuring calcium elevation and mechanical properties of cells [51,52,53,54,55]. By integrating the capabilities of both cell imaging and acoustic tweezer into a single UHF transducer, we demonstrate the feasibility of performing non-invasive trapping and high-resolution imaging of single cells and organelles in a dual mode. The biomechanical properties of cells, as evaluated through scattering and attenuation of sound waves, is determined by the feasibility of performing both trapping and high-resolution imaging.

## 2. Materials and Methods

### 2.1. UHF Transducers

Customized UHF transducers were developed and fabricated previously in our laboratory [49,56]. A 36° rotated Y-cut lithium niobate (LiNbO_3_) single crystal (Boston Piezo-Optics, Bellingham, MA, USA) was used at 110 and 410 MHz. Optimized aperture size and thickness of layers including the piezoelectric layer and the matching layer were designed with the Krimholtz, Leedom, and Matthaei model (PiezoCAD, Sonic Concepts, Bothell, WA, USA). Thickness of each material used for the two transducers is shown in Table 1.

As shown in Table 1, the material types and the number of layers are the same for both transducers. Fabrication procedures were: (1) preparing the backing layer; (2) attaching LiNbO_3_ to the backing layer; (3) lapping both piezoelectric material and backing layer; (4) housing fabrication; (5) assembling; (6) press-focusing; and (7) parylene coating. More details on the transducer design and fabrication procedure can be found in Lim et al. [49,56]. The f-number was designed at 1.3 for both transducers.

**Figure 1:**
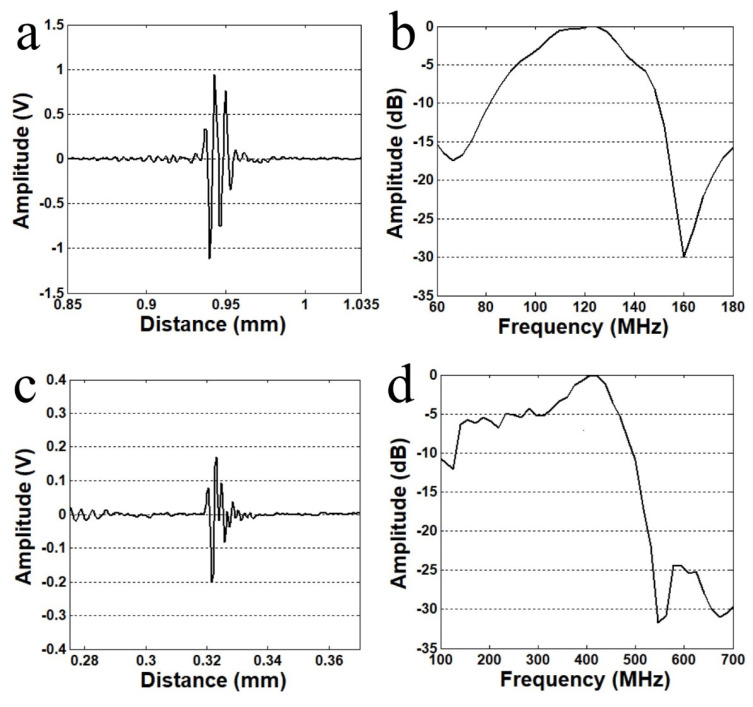
Pulse–echo measurements of fabricated transducers. (**a**) Echo response of 120 MHz transducer, (**b**) frequency spectrum of 120 MHz transducer, (**c**) echo response of 410 MHz transducer, and (**d**) frequency spectrum of 410 MHz transducer.

The performance of the fabricated transducer was measured with a pulse–echo test. The transducer was driven by a pulser/receiver (DPR500, JSR Ultrasonics, Pittsford, NY, USA). Acoustic input parameters were: pulse repetition frequency (PRF) of 200 Hz, 50 V damping, and 2.3 μJ energy per pulse. An X-cut quartz target was used as the reflector. Echo signal was achieved at focal distance. Figure 1 shows pulse–echo measurements of 120 MHz and 410 MHz transducers. The −6 dB fractional bandwidth was 50 and 31% for 120 MHz and 410 MHz transducers, respectively.

**Figure 2:**
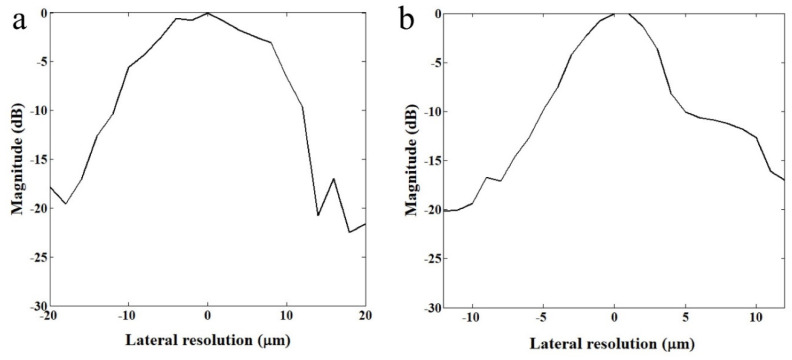
Relative magnitude distributions in lateral direction. (**a**) 110 MHz and (**b**) 410 MHz transducers.

The lateral resolutions of two transducers were measured with a wire target. The 2.5 μm tungsten wire (California Fine Wire, Grover Beach, CA, USA) was immersed in water and scanned in lateral direction at a resolution of 0.5 μm. The reflected echo signal was constructed to demonstrate the lateral resolution of each transducer. Figure 2 shows −6 dB beam widths of 110 MHz and 410 MHz transducers were measured to be 20 μm and 6.5 μm, respectively.

### 2.2. Experimental Setup

**Figure 3:**
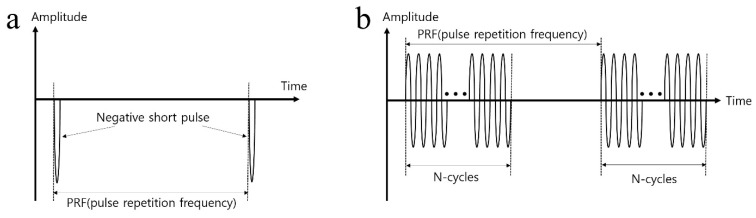
(**a**) Excited negative short pulse waveform for cell-imaging and (**b**) multiple cycles of signal waveform for acoustic tweezer.

**Figure 4:**
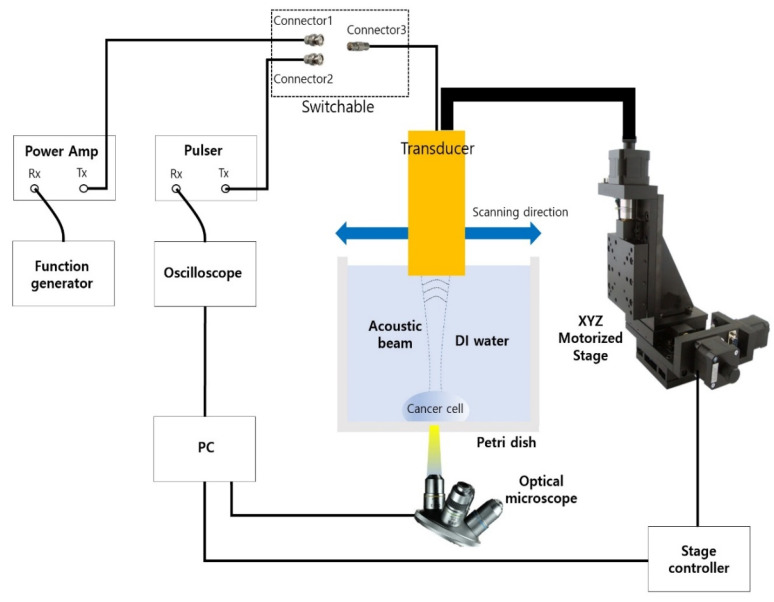
Experiment setup for B-mode cell imaging and acoustic tweezer.

In order to generate the acoustic power for two modes of ultrasonic imaging and acoustic tweezers, different types of waveform are required as shown in Figure 3. For an imaging application, a negative short pulse, which is generated from the pulse’s transmitter, was used because it covered a wide 3-dB bandwidth. On the other hand, multiple cycles of the waveform to the transducer were applied since high pressure or acoustic energy was necessary for continuous manipulation of objects for an acoustic tweezer experiment. Figure 4 demonstrates the experiment setup for B-mode imaging and acoustic tweezers. For two experiments, we used a function generator (AFG3102, Tektronics, Beaverton, OR, USA), a power amplifier (10U1000, AR Inc., Souderton, PA, USA), an oscilloscope (Waverunner 204Xi, Lecroy, Chestnut Ridge, NY, USA), a pulser (JSR Ultrasonics DPR500, Imaginant, Pittsford, NY, USA), a motorized stage (SGSP33-200, Optosigma Corporation, Santa Ana, CA, USA), an optical microscope (IX-71, Olympus, Center Valley, PA, USA), and a CMOS camera (ORCA-Flash2.8, Hamamatsu, Japan).

Since one cycle or short pulse was required for a cell imaging experiment, connectors 2 and 3 were connected for the imaging experiment. The pulse Tx/Rx with an operating frequency of up to 500 MHz was controlled by a PC. Furthermore, the PC could adjust the motorized XYZ stage by 1 μm steps and show the image from the optical microscope. For the experiment, cancer cell’s location was monitored through an optically transparent petri dish with the microscope set to the bottom. Finally, a B-mode image was shown after signal and image processing. MATLAB was used for the processing. For the acoustic tweezer experiment, connectors 1 and 3 were connected. A function generator and a 50 dB power amplifier were also used. In this case, the number of cycles for PRF of 1 kHz was 4100.

### 2.3. PC-3 Preparation

For a cell imaging experiment, a highly invasive human prostate cancer cell (PC-3) line was used. PC-3 cells were purchased from ATCC (Manassas, VA, USA) and maintained in complete growth medium which consisted of ATCC formulated F12-K medium (Kaighn’s modification) supplemented with 10% *v*/*v* fetal bovine serum (FBS) and 100 U/mL penicillin/streptomycin. Cells were seeded at a density of 1.3 × 104 cells/cm^2^ and cultured in 5% CO_2_ at 37 °C. A trypsin-ethylenediaminetetraacetic acid (trypsin-EDTA) solution was purchased from Invitrogen (Invitrogen, Grand Island, NY, USA) and used for cell suspension. Hank’s balanced salt solution (HBSS) was purchased from Invitrogen (Grand Island, NY, USA) for maintaining cells during the cell experiment. After suspending the cell from the petri dish, HBSS was used to neutralize the trypsin-EDTA for the cell safety.

## 3. Results

### 3.1. B-Mode Imaging of a Single Cell and Particle Using UHF Transducers

**Figure 5:**
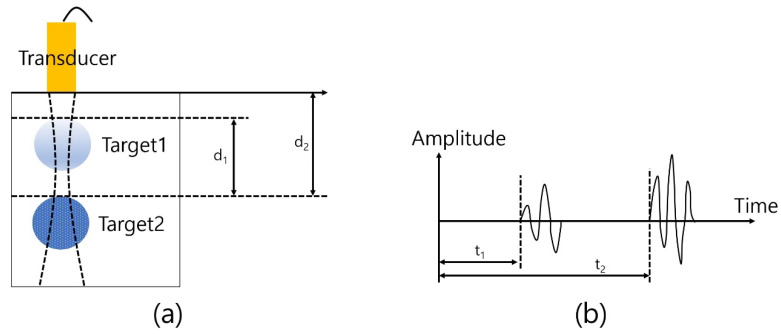
Principle of ultrasound imaging: (**a**) Target and transducer and (**b**) received signal from targets.

The basic principle of ultrasound imaging is shown in Figure 5. The transducer generates an acoustic propagation wave to the front and the reflected and backscattered signal travels back to the transducer as the propagation is processed. A certain sound speed is dependent on the medium or matter and there is a time difference between the signals from target 1 and target 2. Specifically, target 1 is relatively closer than target 2 to the transducer and the difference results in the time difference between t1 and t2. Also, the target’s properties, which include stiffness, sound speed, and attenuation coefficient determine the reflected signal’s amplitude so that it can be converted to the B (brightness)-mode image after signal processing, such as filtering, envelope detection, and log compression.

**Figure 6:**
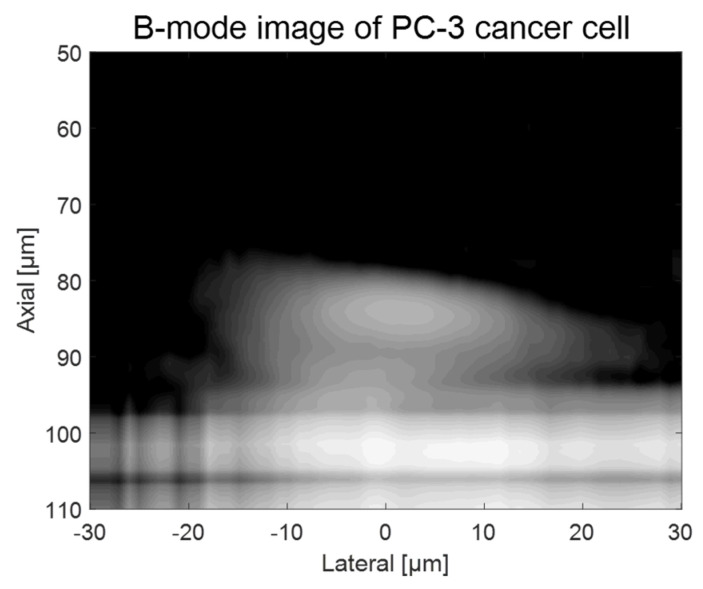
B-mode imaging of a PC-3 cell with a 110 MHz transducer.

**Figure 7:**
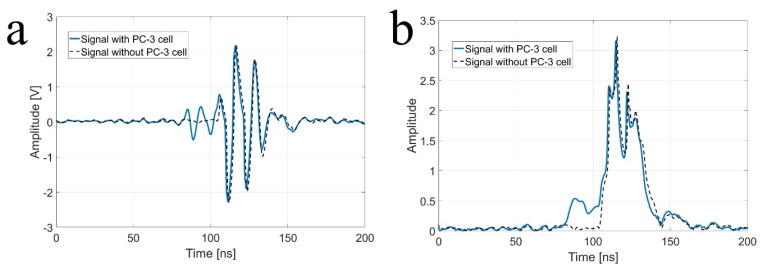
(**a**) Raw signal from the center with and without (*w/o*) a PC-3 cell with a 110 MHz transducer, and (**b**) signal after Hilbert transform from the center with and *w/o* a PC-3 cell with a 110 MHz transducer.

As shown in Figure 2, a custom-fabricated transducer can lead to a lateral resolution of 20 μm with a 110 MHz transducer and a lateral resolution of 6.5 μm with 410 MHz transducers. A single-cell imaging requires spatial resolution with a high operating frequency. Because the diameter of a PC-3 cell was quite large (>32 μm) compared to the measured acoustic beam width, the scanning range was set to 60 μm with 1 μm step, covering the whole single cell. A B-mode image of a PC-3 single cell with a 110 MHz transducer is shown in Figure 6. Additionally, the radio frequency (RF) signals with and without (*w/o*) a cell are plotted in Figure 7a, and the data-processed signal with Hilbert transform from the center with and *w/o* a cell are displayed in Figure 7b. There was a difference in the amplitude *w/o* a cell around 80~100 μs. The strong signal after 100 ns was from the surface of the peri dish. Based on measured data, it was enough to see the cell’s shape, at least at the cell’s surface. Thus, we could estimate the cell’s height to be about 20 μm with a width of around 45 μm.

**Figure 8:**
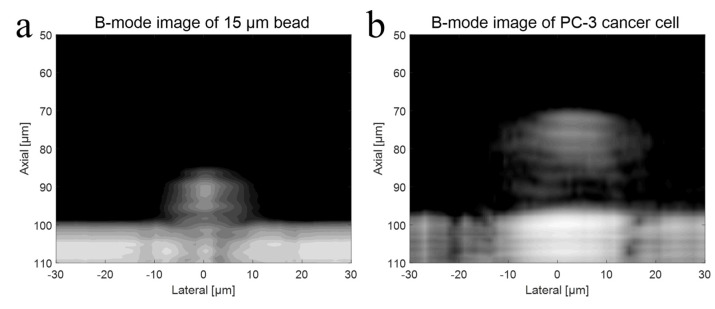
(**a**) B-mode imaging of a 15 μm bead with a 410 MHz transducer, and (**b**) B-mode imaging of a PC-3 cell with a 410 MHz transducer.

**Figure 9:**
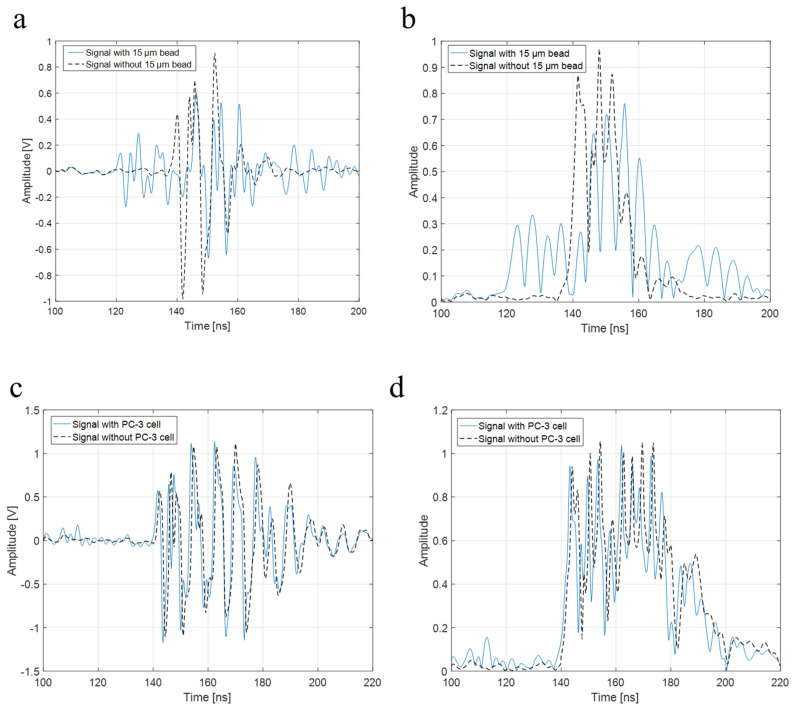
(**a**) Raw signal from the center with and *w/o* 15 μm bead with a 410 MHz transducer, (**b**) signal after Hilbert transform from the center with and *w/o* 15 μm bead with a 410 MHz transducer, (**c**) raw signal from the center with and *w/o* a PC-3 cell with a 410 MHz transducer, and (**d**) signal after Hilbert transform from the center with and *w/o* a PC-3 cell with a 410 MHz transducer.

To verify the higher operating frequency’s advantage, a bead with a diameter of 15 μm and a PC-3 cell were used in the experiment with a 410 MHz transducer. The B-mode images of a 15 μm bead and a PC-3 cell with a 410 MHz transducer were acquired, respectively, as shown in Figure 8a,b. Further, Figure 9a represents raw signals from the center with and *w/o* a 15 μm bead with a 410 MHz transducer, and Figure 9b displays the signal after Hilbert transform from the center with and *w/o* a 15 μm bead with a 410 MHz transducer. For more detailed analysis of a single-cell imaging, raw signals and the data processed signals with and *w/o* a cell were plotted in Figure 9c,d, respectively.

### 3.2. A Sinlge-Cell and Particle Trapping Using Acoustic Tweezers

**Figure 10:**
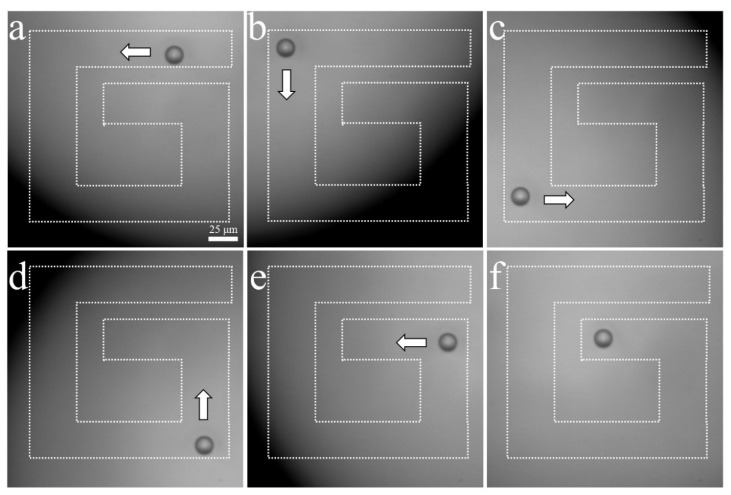
Microscopic photographs of a 15 μm polystyrene bead manipulation using a 410 MHz transducer: (**a**–**f**) show the movement of a bead along the SBAT direction. The white arrow represents the direction of transducer movements. A bead is moved through a “G” pattern. Scale bars indicate 25 μm.

**Figure 11:**
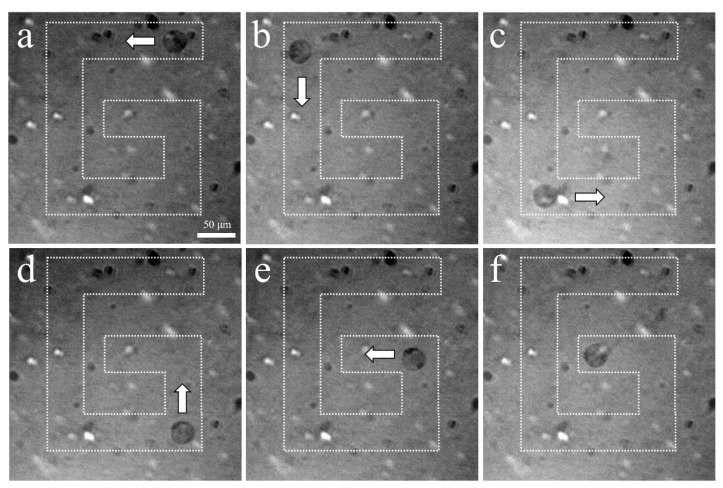
Microscopic photographs of a single PC-3 trapping using a 410 MHz transducer: (**a**–**f**) show the movement of a cell along the SBAT direction. The white arrow represents the direction of transducer movements. PC-3 is moved through a “G” pattern. Scale bars indicate 50 μm.

The experimental arrangement for acoustic tweezers is shown in Figure 4. A function generator was connected to a 50 dB power amplifier and 410 MHz sinusoidal burst signals were driven to the transducer. The transducer was mounted on a three-axis motorized translation linear stage for mechanical movement. The movement of a single cell was observed using an inverted microscope and recorded via a CMOS camera. To demonstrate single-cell manipulation in two dimensions, the transducer was driven using the following acoustic parameters: a driving frequency of 410 MHz, a duty factor of 1%, a PRF of 1 kHz, and a peak-to-peak input voltage (V_pp_) of 7.94. Figure 10 and Figure 11 show the motion of a 15 μm bead and a single cell manipulated, respectively. Figure 10a–f and Figure 11a–f show the movement of a bead and a cell, respectively, along the SBAT direction. The white arrow shows a single cell’s movement along the SBAT direction to write the letter “G”.

## 4. Discussion

### 4.1. A Single-Cell B-Mode Imaging

When a wave of acoustic propagation encounters a cell, the scattering phenomenon will vary based on the cell’s properties, such as size, surface, and shape. These properties can impact the scattered signal produced by the propagation wave. Therefore, it is important to examine the backscattered signal, as it reflects the specific response of the cell. To achieve accurate signal analysis, it is necessary to obtain the echo signals from the cell as unadulterated as possible. The use of a UHF transducer is critical in this regard, as its ultrahigh operating frequency enables it to differentiate between the cell’s signal and that of the petri dish. Theoretically, a shorter wavelength would result in better resolution, reducing distortion caused by overlapping echoes from the cell and petri dish.

B-mode imaging can be used to study the cell’s stiffness and deformability by processing the signal, using techniques such as filtering, envelope detection, and log compression. Scientific principles dictate that shorter wavelengths should lead to higher resolution, resulting in a clearer image of the cell’s width and height. To image a single cell with a diameter of 20–30 μm, the center frequency of the transducer should be higher than 100 MHz.

The shape of bead was clearly depicted as spherical in the image, and its size was accurately represented following image processing. However, if the frequency of the transducer was inadequate for the experiment, the size of the target would appear larger after data processing. The cell’s B-mode data were acquired using a 410 MHz transducer. Theoretically, a UHF transducer would result in better resolution and provide more detailed information about the cell, as a shorter wavelength results in better spatial resolution. The images revealed the cell nucleus more clearly compared to the 110 MHz experiment. This allowed for a precise estimation of the cell’s boundary and shape. If the wavelength of the transducer is larger than the height of the cell, the signals from the cell membrane and nucleus may interfere with each other, making it difficult to analyze the difference in signals.

According to the specifications of the transducer, the wavelength of the 410 MHz transducer is 3.7 μm, while that of the 110 MHz transducer is 12.5 μm. This means that the 410 MHz wave can penetrate a single cell more frequently, leading to more precise results in the image. Thus, it can be concluded that an ultrahigh operating frequency is an indispensable tool for cell imaging and is beneficial in detecting specific signals from a single cell.

If a conventional low-operating-frequency transducer (<20 MHz) is used, it will limit the lateral and axial resolution to several hundred micrometers and is unsuitable for the cell imaging application. On the other hand, gigahertz frequency transducers offer high resolution imaging of the cell layer and nucleus, but they face the challenge of high attenuation and limited penetration depth. Hence, selecting the appropriate transducer frequency is crucial. In addition, the small size of the cell and the weak received signal compared to that from quartz pose challenges in cell imaging, due to the similar acoustic impedance of cells and water. To overcome these limitations, highly focused (low f#) transducers with high sensitivity were used in this experiment, which allowed for high spatial resolution less than the target size. This resulted in the successful generation of detailed information from a PC-3 cell, enabling the analysis of the cell’s properties, such as stiffness and invasiveness. For future studies, additional techniques such as integrated backscattering coefficients, attenuation slopes, and y-intercepts at 0 Hz in both the frequency and time domains can be applied for more detailed property information. The amplitude of the reflected echo signals in the time domain is dependent on the mechanical properties of the target, and there is typically greater attenuation at higher frequencies than at lower frequencies, proportional to the frequency. Thus, spectral analysis such as integrated backscattering coefficients and attenuation slopes can provide additional information in the frequency domain and differentiate cells from beads, even if their echo signal amplitudes are similar or close to each other due to their similar size. Furthermore, while this study employed B-mode imaging with 1-D imaging shown, 2-D sectional imaging and acoustic impedance imaging are possible with additional mechanical scanning and signal processing.

### 4.2. Acoustic Tweezer of a Sinlge Cell

Acoustic tweezers are an ultrasonic technology used to manipulate the position and movement of bioparticles ranging in size from nanometer-sized extracellular targets to millimeter-sized multicellular organisms. The ultrasonic wave creates a vortex-shaped beam profile that traps the target at the center. The technology has advanced from single particle trapping to precise two-dimensional and three-dimensional rotation control of cells. Acoustic tweezers offer the advantages of being contactless and label-free, as well as not causing thermal damage, unlike optical tweezers. However, there is a limitation in the beam width due to transducer specifications such as operating frequency and f#, which makes it challenging to use for single-cell manipulation. To address this, in this study, we used UHF transducers with a highly focused method to achieve single-cell trapping.

Besides conducting cell imaging studies, the performance of an acoustic tweezer using an identical transducer was also demonstrated. The use of UHF transducers resulted in a finer beam width compared to low operating frequencies, providing a lateral resolution of 6.5 μm, which is significantly less than the diameter of a single cell. This made it possible to manipulate individual cells more precisely. It should be noted that this is the first time that an individual spherical suspended cell has been manipulated using a UHF transducer with a frequency above 400 MHz. As a next step, the physical modulus of the cell will be investigated through acoustic tweezers and the biomechanical properties of the cell will be evaluated through the scattering and attenuation of sound waves.

Additionally, UHF transducers have the advantage of not causing thermal damage to the target cell, unlike optical tweezers, which can cause thermal damage due to the use of laser beams. As a result, optical tweezers often manipulate beads attached to the cell, rather than the cell itself. Figure 11 demonstrates that the UHF acoustic tweezers can trap individual cells directly without causing thermal damage. The trapping force generated by UHF transducers can vary from pico- [56] to nanonewtons [49], depending on input acoustic parameters. This strong trapping force has the potential to enable the trapping of cell clusters in the future, beyond just single cells. However, a limitation of UHF transducers is the difficulty in measuring their actual pressure and intensity, as commercial hydrophones are typically limited to frequencies of less than 60 MHz. If hydrophone systems could support UHF transducers, it would become easier to trap micro-sized particles and cells with greater precision. These calibrated data would allow for the expansion of acoustic tweezer applications, particularly in single-cell analysis.

## 5. Conclusions

This paper proposes the use of UHF transducers for cellular applications. To support this proposition, LiNbO_3_ piezo-material UHF single-element transducers were utilized, and two applications were experimentally demonstrated using PC-3 cells and a 15 μm sphere-shaped bead. The results of the cell imaging system support the hypothesis by successfully detecting signals from a cell and imaging both a cell and bead on a petri dish using an acoustic scanning method. Previous research relied on high-frequency transducers for stimulation and required an optical microscope to track and monitor cell movements or changes as low-frequency transducers were inadequate for ultrasound cell imaging. The UHF transducer used in this study, however, provides both imaging and treatment capabilities using a single ultrasonic device and system.

In conclusion, the experiments using the home-made dual-mode UHF transducer showed exceptional acoustic trapping performance and high-resolution cell imaging capabilities, demonstrating the possibility, extensibility, and utility of UHF transducers as a valuable tool in biomedical research. Future studies should investigate the properties, apoptosis, and growth of cells based on received signals in the time and frequency domains using UHF ultrasound.

## Figures and Tables

**Table 1:** Design parameters for UHF single-element transducers.

Layer	Material	Thickness	
		110 MHz	410 MHz
Piezoelectric layer	LiNbO_3_	26 μm	7.3 μm
Matching layer	Parylene	5.2 μm	1.1 μm
Backing layer	E-Solder 3022	1000 μm	1000 μm

## Data Availability

Not applicable.
